# SARS-CoV-2 S2P spike ages through distinct states with altered immunogenicity

**DOI:** 10.1016/j.jbc.2021.101127

**Published:** 2021-08-27

**Authors:** Adam S. Olia, Yaroslav Tsybovsky, Steven J. Chen, Cuiping Liu, Alexandra F. Nazzari, Li Ou, Lingshu Wang, Wing-Pui Kong, Kwan Leung, Tracy Liu, Tyler Stephens, I-Ting Teng, Shuishu Wang, Eun Sung Yang, Baoshan Zhang, Yi Zhang, Tongqing Zhou, John R. Mascola, Peter D. Kwong

**Affiliations:** 1 Vaccine Research Center, National Institute of Allergy and Infectious Diseases, National Institutes of Health, Bethesda, Maryland, USA; 2 Vaccine Research Center Electron Microscopy Unit, Cancer Research Technology Program, Leidos Biomedical Research Inc, Frederick National Laboratory for Cancer Research, Frederick, Maryland, USA

**Keywords:** COVID-19 vaccine, pH-induced folding, SARS-CoV-2 spike, S2P, unfolding, BLI, bio-layer interferometry, cryo-EM, cryo-electron microscopy, CTF, contrast transfer function, DSC, differential scanning calorimetry, EM, electron microscopy, NS-EM, negative-stain electron microscopy, NTD, N-terminal domain, PBS, phosphate buffered saline, RBD, receptor-binding domain

## Abstract

The SARS-CoV-2 spike is the primary target of virus-neutralizing antibodies and critical to the development of effective vaccines against COVID-19. Here, we demonstrate that the prefusion-stabilized two-proline “S2P” spike—widely employed for laboratory work and clinical studies—unfolds when stored at 4 °C, physiological pH, as observed by electron microscopy (EM) and differential scanning calorimetry, but that its trimeric, native-like conformation can be reacquired by low pH treatment. When stored for approximately 1 week, this unfolding does not significantly alter antigenic characteristics; however, longer storage diminishes antibody binding, and month-old spike elicits virtually no neutralization in mice despite inducing high ELISA-binding titers. Cryo-EM structures reveal the folded fraction of spike to decrease with aging; however, its structure remains largely similar, although with varying mobility of the receptor-binding domain. Thus, the SARS-CoV-2 spike is susceptible to unfolding, which affects immunogenicity, highlighting the need to monitor its integrity.

SARS-CoV-2, the etiological cause of the COVID-19 pandemic, has been the focus of a worldwide effort both to understand its biology and to develop clinical products to treat or prevent infection (1, 2, 3, 4). A major focus of SARS-CoV-2 research involves the trimeric spike glycoprotein, a type-1 fusion machine responsible for receptor engagement and viral entry into target cells (5, 6, 7, 8, 9). Antibodies targeting the receptor-binding domain (RBD) as well as the N-terminal domain (NTD) have been identified from convalescent donors that potently neutralize SARS-CoV-2 (10, 11, 12, 13, 14, 15, 16, 17, 18), and several of these have been shown clinically to ameliorate infection (19, 20).

As is the general case for type-1 fusion proteins, the mature spike is embedded in the viral membrane in a metastable prefusion conformation, which undergoes large structural rearrangements as it transitions to its postfusion form, a transition that facilitates merging of viral and target cell membranes and virus entry into the cell. Two proline mutations, originally developed for the MERS coronavirus spike (21), have been shown to stabilize the soluble trimeric ectodomain in its prefusion conformation. When combined with the T4-bacteriophage foldon trimerization domain and a mutated furin site to prevent cleavage (9), the “S2P” construct has become the *de facto* protein format for research into the biological function of the SARS-CoV-2 spike. Related di-proline constructs have been utilized for approved Moderna and Pfizer mRNA vaccines (22, 23) as well as for the proteinaceous Sanofi and Novavax vaccines, and the spike component of the Johnson & Johnson adenovirus-based vaccine (24, 25, 26). While further stabilized constructs have been developed, including Hexapro with six proline mutations (27), the soluble, trimeric S2P remains widely studied and utilized.

The effect of refrigerated storage on S2P has been documented as resulting in global unfolding, which can be counteracted by a heat-shock procedure and potentially mitigated by low-pH storage (28). However, details of unfolding and its impact on S2P immunogenicity—especially the elicitation of neutralizing responses—have remained unclear. Here, we investigate the cold storage-induced unfolding of S2P by measuring both its antigenicity and its immunogenicity and by comparing negative-stain electron microscopy (EM) images and cryo-EM structures of fresh and aged S2P. Notably, upon cold storage for 30 days, S2P still elicits substantial ELISA responses in mice, but no longer elicits neutralizing responses. Further, we demonstrate that a simple low-pH treatment procedure reverses the storage-induced unfolding.

## Results

### SARS-CoV-2 S2P unfolds through multiple states at 4 °C and physiological pH

**Figure 1:**
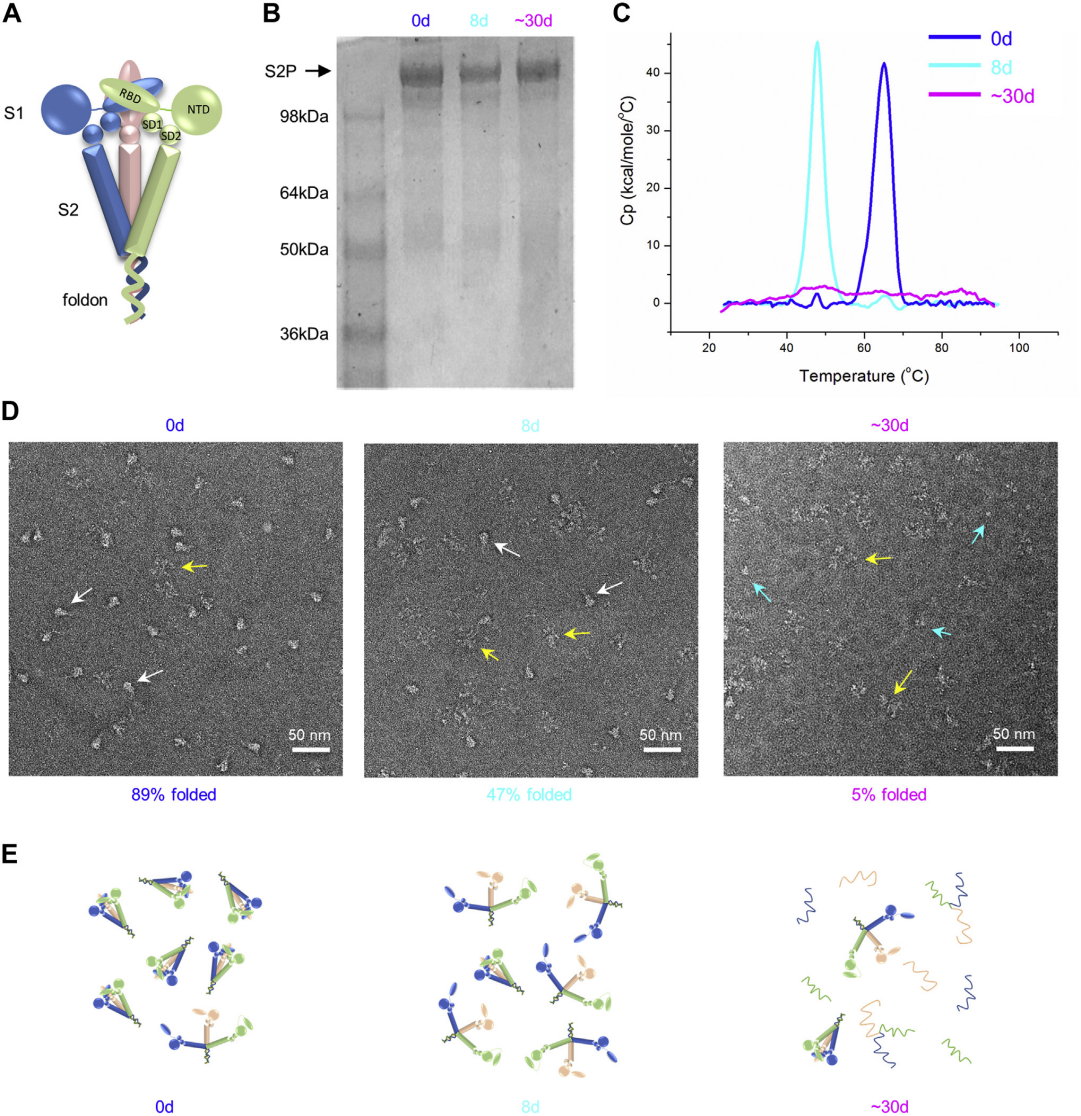
**SARS-CoV-2 spike S2P unfolds through multiple states at 4 °C.***A*, schematic representation of fully folded S2P spike. Domains of the S1 subunit are labeled. *B*, SDS-PAGE of S2P samples, fresh (0 day) and after storage at 4 °C in PBS for 8 days and ∼30 days demonstrating that the S2P spike polypeptide remained intact after storage. *C*, differential scanning calorimetry (DSC) measurements of the S2P samples. *D*, NS-EM images of 0-day, 8-day, and ∼30-day aged S2P samples. Fresh S2P appeared mostly as trimeric protein, 8-day aged S2P appeared as a mixture of intact trimers and unfolded molecules retaining their trimeric state, and 30-day aged S2P contained mostly unfolded or dissociated protein. Folded fraction quantifications are based on averages from 25 micrographs (see Experimental procedures). *White*, *yellow*, and *cyan arrows* point to folded trimers, unfolded trimers, and small fragments of S2P spike, respectively. *E*, *left* to *right*, schematic for stages of cold-induced unfolding. Freshly purified protein (0 day) contains fully folded spike trimers. Short-term storage (8 days) induces weakening of interprotomer contacts, with the trimer held together by the C-terminal foldon. Prolonged storage (∼30 days) yields mostly unfolded and dissociated protein.

The S2P spike glycoprotein (Fig. 1*A*) was expressed in mammalian cells and purified using metal affinity and size-exclusion chromatography, following the general protocol established previously (9), maintaining the protein at neutral pH and room temperature. Following purification, the protein was stored at 4 °C, to mimic typical storage conditions. We then investigated this S2P construct at several time points to determine the stability of the protein as a function of storage time. Notably, storage for more than 30 days at 4 °C in phosphate buffered saline (PBS) did not cause cleavage or other major peptide modifications to the protein, as indicated by SDS-PAGE (Fig. 1*B*). However, differential scanning calorimetry (DSC) showed that storage at 4 °C in PBS for 8 days resulted in a drop of melting temperature from 65.1 °C for freshly purified protein to 47.9 °C, although the overall energy of unfolding, as calculated from the peak area, remained similar at ∼250 kcal/mol (Fig. 1*C*). This 8-day aged S2P sample also exhibited changes in the conformational state as detected by negative-stain electron microscopy (NS-EM) (Fig. 1*D*). While molecules of freshly purified S2P appeared primarily as compact, trimeric particles, unfolded species prevailed in the 8-day aged S2P, with the estimated fraction of compact trimeric particles reduced from 89% to 47%. It is likely that the NS-EM captured the states of the protein in equilibrium between the folded and unfolded conformations, with individual domains still intact in the unfolded spike.

After approximately 1 month of storage in PBS at 4 °C, the aged S2P (∼30 days) appeared fully unfolded. This was indicated by the lack of a DSC transition (Fig. 1*C*) and near complete disappearance of compact trimeric particles in the NS-EM images (estimated fraction of folded molecules: 5%) (Fig. 1*D*). In addition to the unfolding of S2P spike trimers, the appearance of smaller particles was also noticed, suggesting complete dissociation of the trimer. Overall, the results indicate that the SARS-CoV-2 S2P trimer is unstable when stored in PBS at 4 °C.

### Low pH exposure refolds 8-day aged S2P spike protein

**Figure 2:**
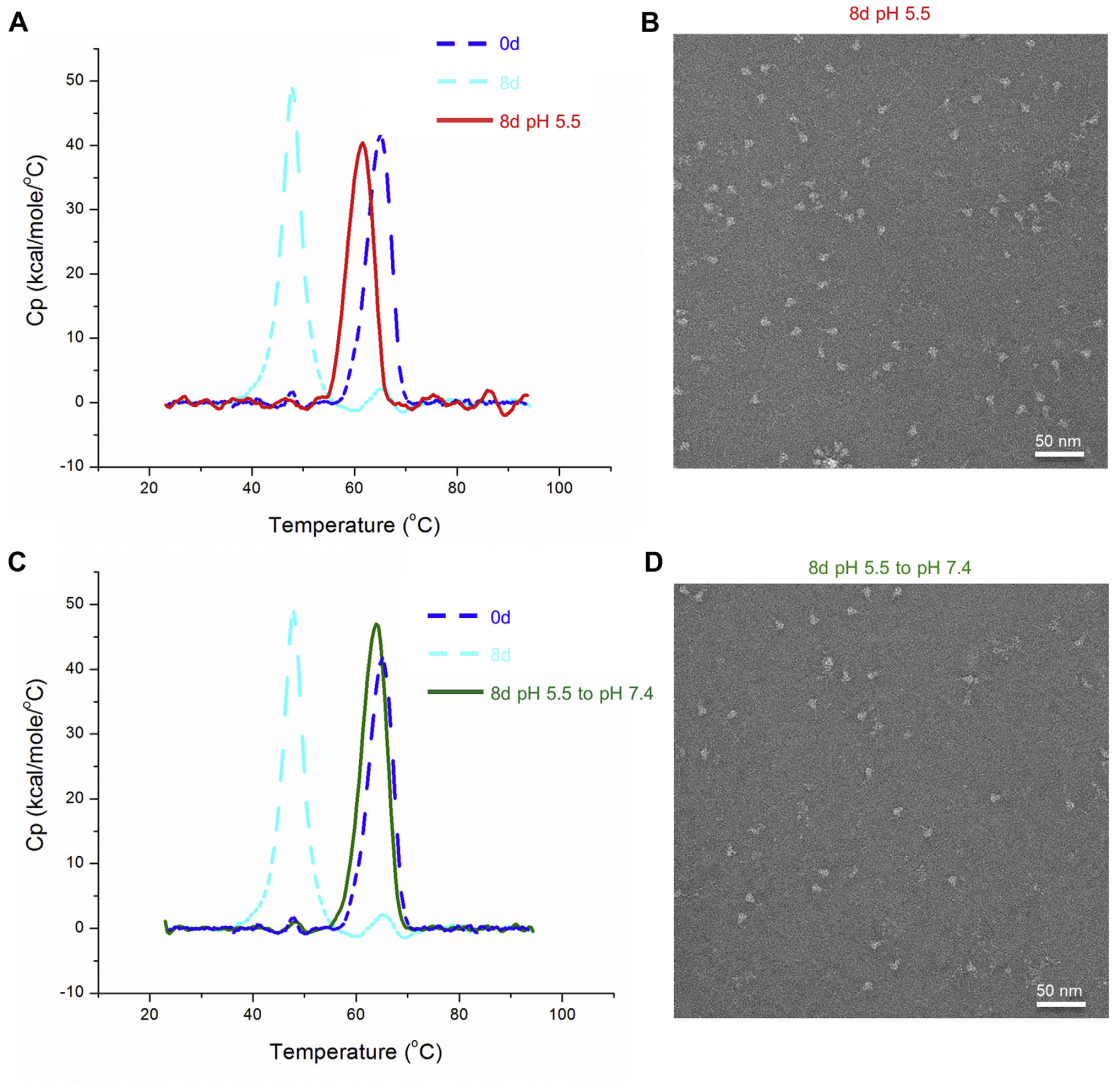
**Low pH refolds 8-day aged SARS-CoV-2 S2P spike.***A*, DSC melting curve of 8-day aged spike after buffer exchange into pH 5.5 (*red solid line*), compared with those of the 0-day sample (*blue dashed line*) and 8-day aged spike (*cyan dashed line*). The low-pH-treated protein reverted to its folded state in low pH. *B*, NS-EM image of the 8-day aged sample after buffer-exchange into pH 5.5. The spikes were well-folded and trimeric, similar in appearance to the 0-day sample. *C*, DSC scan of the 8-day aged sample after treatment at pH 5.5 and then returning to pH 7.4 (*green solid line*), compared with those of the 0-day sample (*blue dashed line*) and 8-day aged spike (*cyan dashed line*). The low-pH-treated spike retained its folded characteristic when returned to neutral pH. *D*, NS-EM image showing that the spike remained folded after low-pH treatment followed by transferring back to neutral pH.

One potential mechanism for the observed loss of the ordered trimeric S2P structure was that the trimeric core of the protein disassembles during storage, with the individual domains retaining their structural state and the protomers remaining associated *via* the foldon trimerization domain (Fig. 1*E*). To test this possibility, we subjected the 8-day aged S2P to lower pH conditions, as this was previously observed to stabilize the all RBD-down conformation of the spike (29). We found that transferring 8-day aged S2P protein into a buffer of pH 5.5 for 10 min restored its DSC-melting profile to a shape nearly identical to that of the fresh protein (Fig. 2*A*) and restored a fully folded conformation of compact trimeric particles as revealed by NS-EM (Fig. 2*B*). Similar results could be achieved by exposing the S2P spike to acidic pH in the 4.0 to 5.5 range, whereas treatment at pH 6.0 failed to fully restore the trimer (Fig. S1). Of note, we did not observe unfolding of the S2P trimer by NS-EM when it was stored at 4 °C in pH 4.0 for 23 days (Fig. S2*A*).

To determine if the impact of low-pH-induced refolding could be maintained upon returning the protein to neutral pH, the 8-day aged protein that was subjected to the 10-min low-pH treatment was transferred back into PBS using buffer exchange and then reassessed by DSC and NS-EM. Such pH-treated protein exhibited essentially identical melting temperature to that of the freshly purified protein (Fig. 2*C*) and retained its folded, trimeric conformation (Fig. 2*D*). This effect was also observed by NS-EM when the protein was refolded at pH 4.0 to 5.0 (Fig. S1). Furthermore, subsequent storage at 4 °C in PBS resulted in gradual unfolding similar to that observed in protein samples not subjected to low-pH treatment, indicating that transient exposure to low pH “resets” the S2P spike to its original state (Fig. S2*B*). We attempted the same pH treatments on the ∼30-day aged S2P protein, but were unable to restore the trimeric particle conformation (Fig. S2*C*), indicating that the unfolding of S2P becomes irreversible after longer storage in PBS at 4 °C. Consistent with the above observations, while we confirmed that the 8-day aged protein refolded successfully in response to raising the temperature to 37 °C, as reported by Edwards *et al.* (28), such temperature treatment failed to rescue the ∼30-day aged S2P spike (data not shown). Overall, the S2P protein appeared to be more stable at low pH, with unfolding induced by short-term storage in PBS at 4 °C reversible by low-pH treatment.

### S2P aging after long storage reduces immunogenicity

**Figure 3:**
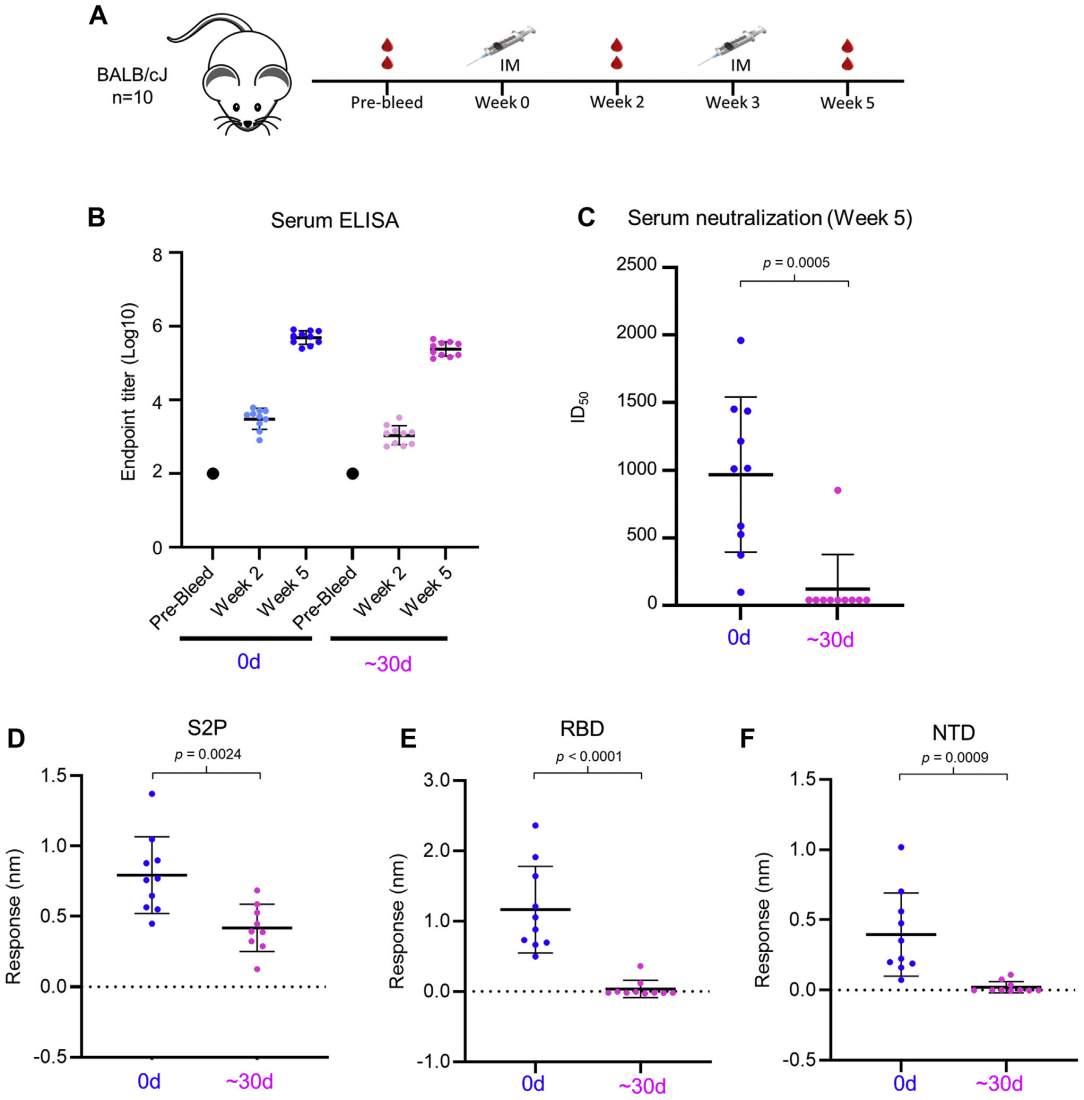
**Aged (∼30 days) SARS-CoV-2 S2P loses capacity to elicit neutralization.***A*, immunization scheme for BALB/cJ mice immunized twice with S2P protein. *B*, ELISA endpoint titers of sera from mice immunized with freshly purified (0 day) S2P spike or spike aged for approximately 1 month (∼30 days) at a dosage of 1 μg. In both groups, similar levels of ELISA titers were observed. A single immunization induced only moderate ELISA signal, but two immunizations elicited strong ELISA signals. *C*, pseudovirus neutralization using immunized mice sera showed a stark contrast between 0-day and ∼30-day protein immunizations, with aged spike giving no neutralization response. *D*–*F*, BLI binding assays of sera from immunized mice at week 5 against immobilized S2P spike (*D*), RBD (*E*), and NTD (*F*).

To address the immunological implications of the aging of the spike S2P protein, we immunized BALB/cJ mice at weeks 0 and 3 with 1 μg of fresh S2P (0 day) or S2P after extended storage in PBS (pH 7.4) at 4 °C for ∼30 days (∼30-day aged) (Fig. 3*A*). No significant difference in serum ELISA titers against S2P was observed between the two groups of mice, and sera from 2 weeks after the last immunization from both groups showed >10^5^ endpoint ELISA titer (Fig. 3*B*). However, serum neutralizing titers against SARS-CoV-2 pseudovirus from these two groups were dramatically different (Fig. 3*C*). Mice immunized with fresh S2P showed SARS-CoV-2 neutralizing titers of ∼1000 mean ID_50_, whereas almost no neutralization was observed with sera from ∼30-day aged S2P-immunized mice.

To gain insight into the lack of correlation between the ELISA titers, which measured the amount of serum antibodies capable of binding S2P, and neutralization titers, which measured the ability of the immunized sera to neutralize SARS-CoV-2, we assessed the mouse sera for reactivity to S2P as well as to isolated RBD and NTD domains (Fig. 3, *D*–*F*, Fig. S3 and Table S1) by Bio-Layer Interferometry (BLI). The sera from the fresh S2P-immunized group bound well to immobilized S2P, RBD and NTD, with an average response between 0.25 and 1.0 nm. However, the sera from ∼30-day aged S2P-immunized animals showed reactivity only to S2P, but no response against the isolated RDB or NTD domains. As most known SARS-CoV-2 neutralizing antibodies target the RBD or NTD regions of the spike, the BLI results indicate that the high ELISA titers, in combination with lack of neutralization from sera of ∼30-day aged S2P immunized mice, could be attributed to elicited antibody responses being directed to nonneutralizing epitopes. Partially corroborating for this attribution is the fact that the single outlier in the ∼30-day aged neutralization, which showed an ID50 of ∼900 (Fig. 3*C*), was the same animal that had a small BLI-measured binding response to RBD (Fig. 3*E*), indicating this animal was able to generate antibodies against the RBD, allowing for the observed neutralization.

### Antigenicity of S2P spike is substantially impacted only after long storage

**Figure 4:**
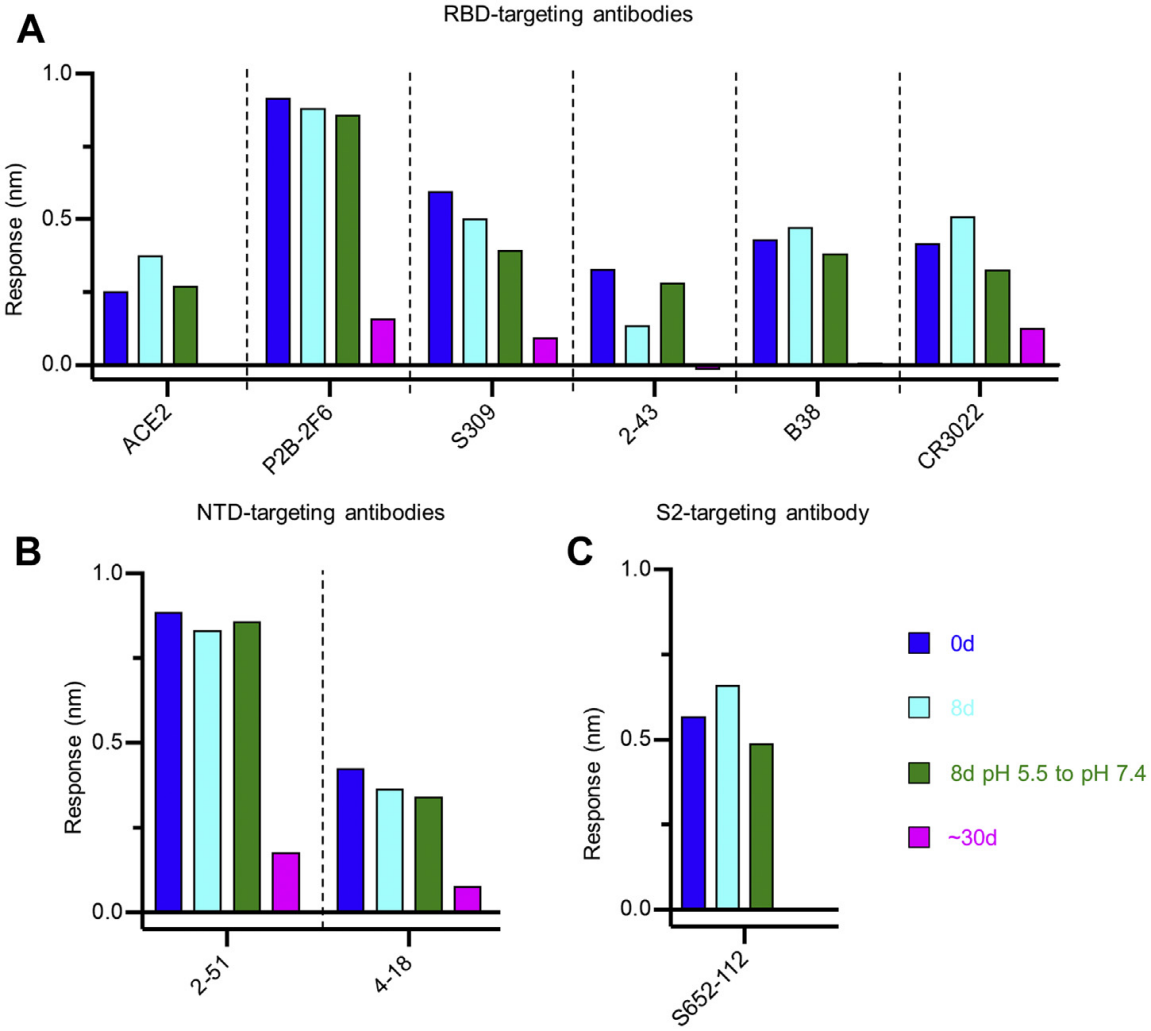
**Antigenicity of SARS-CoV-2 S2P spike is similar between 0 day and 8 days but markedly different for ∼30 days.***A*, BLI measurements of ACE2 and RBD antibodies binding to 25 nM fresh (0 day), aged (8 days, ∼30 days), and low-pH treated spike showed similar binding of 0-day, 8-day, and low pH-treated samples and diminished binding of the ∼30-day sample. This pattern is maintained with antibodies targeting the NTD (*B*) and S2 (*C*) regions of the spike.

To better understand the impact of aging on the epitope presentation of the S2P spike, we performed BLI binding studies using a panel of antibodies and ACE2 to probe recognition of S2P protein of different ages (Fig. 4). The fresh, 8-day aged, and 8-day aged low-pH treated S2P bound well to the immobilized ACE2 receptor, whereas the ∼30-day aged S2P did not bind (Fig. 4*A*). Similarly, in nearly every case the BLI signals were virtually identical between fresh (0 day), 8-day aged, and 8-day aged low-pH treated S2P for binding to RBD-, NTD-, and S2-targeting antibodies (Fig. 4, *A*–*C*). One of the RBD-targeting antibodies, 2-43, had ∼50% decrease in BLI signal for binding to 8-day aged spike relative to the fresh and the low-pH restored samples (Fig. 4*A*), consistent with the fact that the 2-43 epitope is quaternary, and therefore its binding is likely disrupted by trimer unfolding even if the individual domains remain uncompromised (10). As expected, the ∼30-day aged S2P had greatly reduced binding to all the antibodies tested.

These results supported the idea that the individual domains of the spike were largely intact in the 8-day aged sample, and only the quaternary organization of the trimer was affected. By contrast, the structure of the individual domains was likely disrupted in the ∼30-day aged S2P spike, which was completely disordered in solution.

### Cryo-EM reveals increased mobility of the RBDs in the folded fraction of the aged protein in the absence of other structural changes

**Figure 5:**
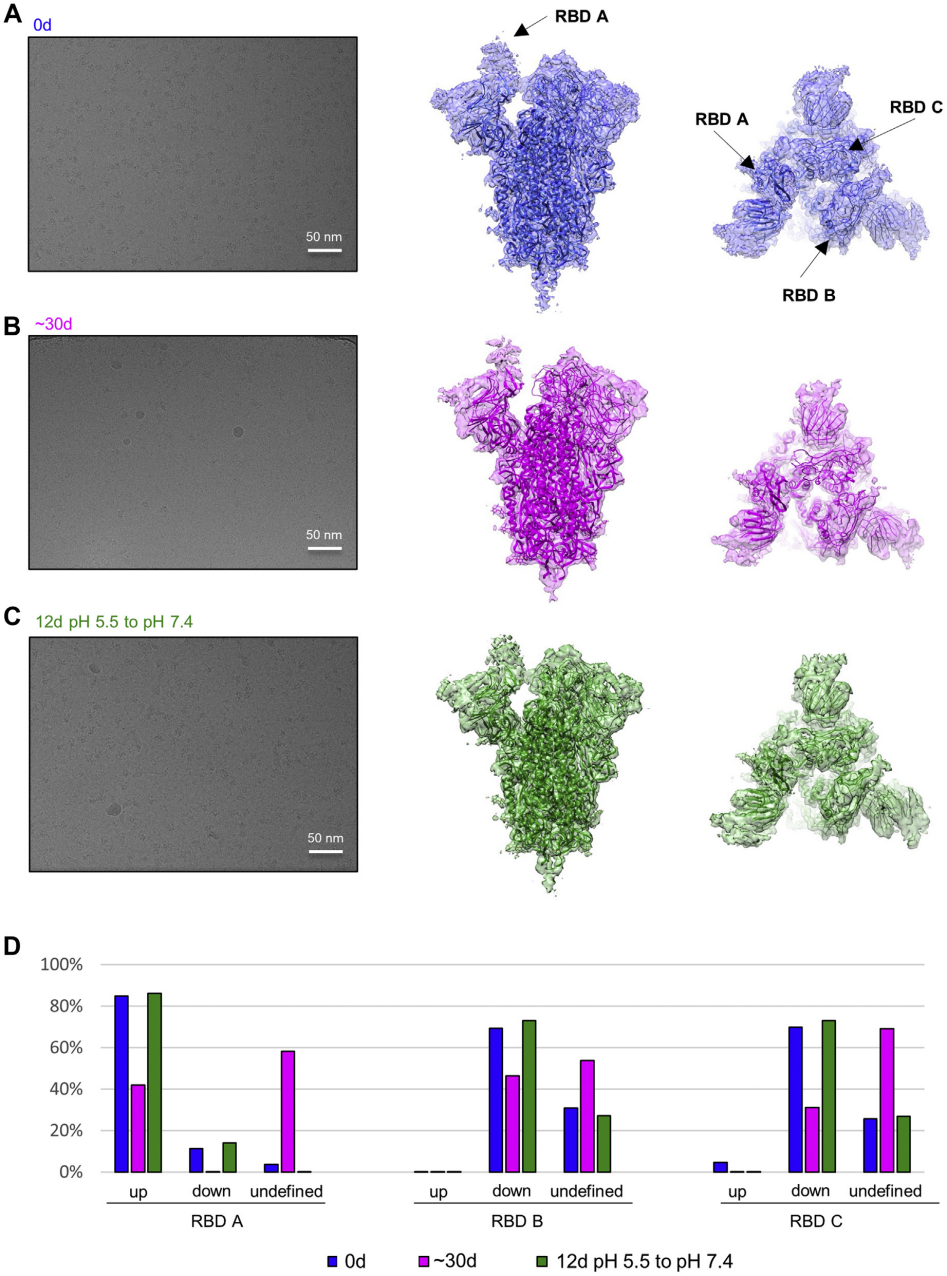
**Cryo-EM reconstructions reveal aged S2P to adopt a similar overall structure, but with fewer ordered particles and alterations in RBD up/down occupancy.***A*, cryo-EM evaluation of S2P protein immediately after purification, with representative micrograph on the *left*, *side view* of the cryo-EM reconstruction in the *middle*, and *top view* on the *right*. *B*, cryo-EM evaluation of S2P protein after storage in PBS at 4 °C for ∼30 days. Far fewer intact particles are present in the representative micrograph. *C*, cryo-EM evaluation of 12-day aged protein subjected to low pH-induced refolding and returned to neutral pH. *D*, quantification of RBDs in the up and down positions, as well as without a defined position, in the three reconstructions presented in (*A*–*C*).

To determine if prolonged storage and transient exposure to mild acidic conditions are associated with structural changes in the folded spike, we used cryo-electron microscopy (cryo-EM) and single particle analysis to solve structures of the spike immediately after purification (0 day), following storage at neutral pH and 4 °C for about 30 days (∼30 days), and after a brief incubation of 12-day aged spike at pH 5.5 followed by returning it to neutral pH (12 days, pH 5.5–7.4). The three cryo-EM specimens were prepared at identical protein concentrations and vitrification conditions, and the same protocol was followed during the computational analysis. We noticed that the number of intact spike molecules per micrograph was substantially reduced for the aged protein *versus* the fresh protein (average particles picked per micrograph using crYOLO software (30): 63 *versus* 243) (Fig. 5), and refolding *via* a transient exposure to pH 5.5 increased the average number of particles per micrograph to 174. While variations in ice thickness and other factors could contribute to the observed differences, these results were consistent with the negative-stain EM data, indicating that the number of compact, fully assembled trimers dropped during storage and could be restored by low-pH treatment. The final cryo-EM datasets for fresh, aged, and refolded spike contained 403,167, 95,641, and 252,067 particles, respectively, and the corresponding maps were refined to resolutions of 2.6 Å, 3.5 Å, and 3.2 Å (Table S2). While there are ten unique combinations of RBD positions for each experimental state (*e.g.*, all down, two down and one undefined, one down and two undefined, etc.), we have only deposited a single structure to represent experimental state, as there were an insufficient number of particles to determine the full ensemble of RBD structures for the aged state, and this variation in RBD conformation is well defined in the published literature (29).

Overall, the structures of the three spike proteins were very similar (r.m.s.d. < 1 Å). However, examination of the maps revealed weaker cryo-EM density for all the RBDs in the “aged” protein in the absence of noticeable differences in other regions (Fig. 5). We quantified the occupancies of the RBDs in the up and down position using 3D classification within a mask encompassing both conformations (Fig. 5*D* and Fig. S4). The position of the RBD was categorized as undefined if the corresponding 3D class did not contain cryo-EM density for this RBD at the set map level (Fig. S4). Such “empty” classes could represent intact but highly mobile RBDs or, alternatively, RBDs that underwent unfolding. We found that, for each RBD, the fractions of the up and down conformations decreased substantially in the aged protein, while the fraction of the RBD without a defined position invariably increased (Fig. 5*D*). Importantly, pH-induced refolding restored the fractions of the up and down conformations to near-original values. Based on these findings, we hypothesize that unfolding of the spike could begin with increased mobility of the RBDs, which could destabilize the S1 subunit of the spike trimer.

## Discussion

While it is expected that long storage of most proteins will result in unfolding and degradation, understanding the rates and pathways of unfolding of the SARS-CoV-2 S2P spike protein and determining the optimal storage conditions can benefit both laboratory investigations and development of clinical products. It was previously reported (28) that cold storage of the S2P spike resulted in unfolding of the protein, with less than 10% of native-like molecules remaining after 7 days at 4 °C, according to NS-EM. Although in our experiments the S2P spike demonstrated slower unfolding (∼50% intact molecules after 8 days), the overall trend was similar. We found that changes in biophysical and structural characteristics of the S2P spike are detectable after just 1 week of storage at 4 °C in neutral pH, the conditions that are often used by default in laboratory research. Moreover, the antigenicity of the S2P protein is affected by 1 month of storage at these conditions, which can have direct implications not only in research but also for development and deployment of protein-based vaccines for COVID-19.

Given that after a week of cold storage the antigenicity of the S2P spike is largely unchanged, yet the thermal stability and assembly as assessed by negative-stain EM are significantly decreased, it is likely that this state of the protein is characterized by weakening of the interprotomer contacts followed by protomer separation, with the trimer being held together by the foldon domain at the C-terminus of the construct, and the RBDs and NTDs remaining intact. Based on the drop in the occupancy of the “up” and “down” conformations of the RBDs detected by cryo-EM in the fraction of aged protein molecules that remained folded, the disassembly process likely begins with increased mobility of the RBDs. This growing mobility weakens the interprotomer interactions, increasing the likelihood of protomer separation. In this regard, we observed earlier that reducing the pH increases the fraction of the RBDs in the down position, with all three RBDs down at pH 4.0 to 4.5 (29). Here we found that prolonged storage of the S2P spike at pH 4.0 prevents unfolding, supporting a link between RBD mobility and trimer stability.

It has been proposed that an ELISA readout could be a useful correlate of vaccine protection (31). In this regard, if only serum ELISA was performed in an immunization experiment with 1-month-old S2P spike, the results could be misleading. While the storage time of 1 month is beyond what most would consider tolerable, it is possible that the loss of the expected correlation between ELISA readout and neutralization potency occurs gradually beyond the 8-day time point as the storage-induced unfolding progresses and becomes irreversible. The aged S2P protein thus provides an example for which ELISA did not predict neutralization, indicating that, while these values are often correlated, it is clearly not always the case.

The S2P construct of the SARS-CoV-2 spike protein is being employed as a clinical product in the Sanofi vaccine candidate (24, 25, 26) as well as a research tool in numerous labs around the world. It is therefore imperative that the quality and state of the protein are well understood during its use. While cold storage-induced denaturation of the S2P spike was reported earlier (28), we show that the unfolding of the S2P protein involves at least two states with distinct biophysical and structural properties and capacities to elicit neutralizing antibody. Furthermore, lowering the pH ameliorates and prevents the degradation of the folded spike trimer, potentially providing a convenient way of long-term storage at 4 °C. As such, if flash freezing and storage at −80 °C are not compatible with study designs or real-world usage, a protein formulation near pH 5.0 allows for refrigerator-temperature storage. Finally, this study highlights the need for thorough biochemical characterization of protein-based immunogens including detailed assessment of stability and structure.

## Experimental procedures

### Expression and preparation of SARS-CoV-2 S2P

SARS-CoV-2 S2P protein was produced as previously described (9). Briefly, 1 l of Freestyle 293-F cells was transfected with 1 mg of SARS-CoV-2 S2P DNA, premixed with 3 ml of Turbo293 transfection reagents. The cells were grown at 37 °C for 5 to 6 days, and then the supernatant cleared by centrifugation and filtration. The supernatant was incubated with nickel resin for 1 h at room temperature, after which the resin was washed with PBS plus imidazole up to 25 mM at pH 7.4. The spike was eluted with 20 mM HEPES pH 7.5, 200 mM NaCl, 300 mM imidazole and concentrated to 2 ml. Following concentration, the protein was applied to a Superdex S-200 column equilibrated in PBS, pH 7.4, at room temperature. The main peak, corresponding to the trimeric protein, was collected, concentrated to 1 mg/ml, and either stored at 4 °C for aging, or flash-frozen in liquid nitrogen and kept at −80 °C for storage.

### Expression and preparation of antibodies

DNA sequences of antibody heavy- and light-chain variable regions of antibodies CR3022 (32), S652-112 (33), B38 (34), 2-4, 4-8, and 2-51 (10) were synthesized and subcloned into the pVRC8400 vector, as described previously (35). Antibody expression was carried out by cotransfection of both heavy- and light-chain plasmids in Expi293F cells (Thermo Fisher) using Turbo293 transfection reagent (Speed BioSystems). On day 5 post transfection, the culture supernatant was harvested and loaded on a protein A column. After washing with PBS for three column volumes, IgG was eluted with an IgG elution buffer (Pierce) and immediately neutralized by addition of one-tenth volume of 1 M Tris-HCl pH 8.0. The antibodies were then buffer exchanged in PBS by dialysis. Dimeric ACE2 (dACE2-Fc) was purchased from Sinobio (Cat# 10108-H02H).

### Differential scanning calorimetry

DSC measurements were performed on the spike samples using a VP-DSC (GE Healthcare/MicroCal). The protein was diluted to 0.25 mg/ml in PBS and measured from 20 to 95 °C at a rate of 1° per minute. Thermal denaturation temperature (T_m_) and enthalpy of unfolding were calculated from fits provided by Origin software.

### Negative-stain electron microscopy

The S2P spike sample was diluted to a concentration of 0.01 to 0.02 mg/ml with buffer containing 10 mM HEPES, pH 7.4, 150 mM NaCl (for samples at neutral pH) or buffer containing 10 mM Na-acetate, 150 mM NaCl (for samples at acidic pH). A 4.7-μl drop of the diluted sample was applied to a freshly glow-discharged carbon-coated copper grid for 15 s. Filter paper was used to remove the drop, and the grid was washed by applying consecutively several 4.7-μl drops of the buffer that was used for sample dilution. After the last drop was removed, three drops of 0.75% uranyl formate were applied in the same manner, and the grid was allowed to dry. Datasets were collected with SerlialEM (36) on an FEI T20 transmission electron microscope equipped with an Eagle CCD camera or with EPU on a ThermoScientific Talos F200C microscope equipped with a Ceta CCD camera. Both microscopes were operated at 200 kV. The pixel size was 0.22 nm and 0.25 nm, respectively. The defocus was set between −0.8 and −1.2 μm. To estimate the fraction of fully folded S2P trimers in freshly purified (0 day) spike and spike stored for 8 and ∼30 days, folded and unfolded trimers were counted manually in 25 micrographs for each sample. A total of 644, 772, and 655 particles were evaluated for the 0-day, 8-day, and ∼30-day S2P spike, respectively.

### Mouse immunization

Animal procedures, housing, and care were performed in accordance with local, state, and federal policies. Mouse experiments were carried out in compliance with regulations and guidelines from the Animal Care and Use Committee of the Vaccine Research Center, National Institute of Allergy and Infectious Diseases, National Institutes of Health (ASP code, VRC-20-0860). Four- to six-week-old female BALB/cJ mice (Jackson Laboratories) were immunized intramuscularly with SARS-CoV-2 S2P glycoprotein (1 μg) adjuvanted with Sigma Adjuvant System (SAS) (Sigma-Aldrich) at weeks 0 and 3. Mice were bled at 2 weeks after each immunization for serological analyses.

### Serum ELISA

The ELISA assays were performed as described (22). Briefly, clear flat-bottom 96-well plates (Thermo Fisher, Catalogue # 442404) were coated with 100 ng of SARS-CoV-2 S-2P without any tags per well in 1× PBS, pH 7.4 at 4 °C for 16 h. After standard washes and 2 h of blocking at room temperature, plates were incubated with serial dilutions of heat-inactivated sera for 1 h at room temperature. Following three washes, the plates were incubated with anti-mouse IgG–horseradish peroxidase conjugate (Invitrogen, Catalogue # G21040) for 1 h at room temperature. After standard washes, the plates were developed using 3,5,3′5′-tetramethylbenzidine (TMB) (KPL) as the substrate to detect antibody responses. The reaction was quenched using 1 N H_2_SO_4_. The plates were read at 450 nm on a SpectraMax Plus plate reader. End-point titers were calculated as the serum dilution that yielded an optical density exceeding 4× background (secondary antibody alone).

### Pseudovirus neutralization assay

SARS-CoV-2 spike pseudotyped lentiviruses that harbor a luciferase reporter gene were produced and neutralization assay was performed as described previously (22, 37). Pseudovirus was produced by cotransfection of 293T cells with plasmids encoding the lentiviral packaging and luciferase reporter, a human transmembrane protease serine 2 (TMPRSS2), and SARS-CoV-2 S (Wuhan-1, Genbank #: MN908947.3). Forty-eight hours after transfection, supernatants were harvested, filtered, and frozen. For neutralization assay serial dilutions of serum (heat activated) were mixed with titrated pseudovirus, incubated for 45 min at 37 °C and added to preseeded 293T-ACE2 cells (provided by Dr Michael Farzan) in triplicate in 96-well white/black Isoplates (PerkinElmer). Following 2 h of incubation, wells were replenished with 150 μl of fresh medium. Cells were lysed 72 h later and luciferase activity (relative light units, RLU) was measured. Neutralization ID50 and ID80 titers were calculated using GraphPad Prism 8.0.2.

### Bio-layer interferometry

Antibody binding to fresh, aged, and pH-shocked SARS-CoV-2 spike was assessed using a FortéBio Octet HTX instrument (FortéBio). Experiments were conducted in tilted black 384-well plates (Geiger Bio-One). PBS +1% BSA was used as buffer. Plates were agitated at a speed of 1000 rpm and kept at 30 °C for the duration of the binding assays. Antibody IgGs (B38, S309, 2-43, CR3022, P2B-2F6, 2-51, 4-18, S652-112) or Fc-tagged dimeric ACE2 (SinoBio) were loaded at 2 to 3 μg/ml onto anti-human Fc biosensors (FortéBio) for 1 min to achieve a capture level of 0.5 to 0.7 nm. The load was consistent with variability between sensors for a given antibody being below 0.13 nm; however, one exception was S309, which loaded ∼0.2 nm higher in the fresh S2P sample than the subsequent S309 trials. Immobilized IgG was dipped into each spike sample (50 nM–3.125 nM). Binding was measured for 5 min and dissociation recorded for 30 min. Octet data analysis software version 12.0 (FortéBio) was used to process the data, perform reference well subtraction to correct for systematic baseline drift, and fit curves. The data was fit using a global fitting and a 1:1 binding interaction model. Plots were generated using GraphPad Prism.

Mouse sera were assessed for binding to SARS-CoV-2 S2P protein, RBD and NTD domain. Biotinylated SARS-CoV-2 S2P protein, RBD and NTD domain were immobilized to SA biosensors, the sensor tips were subsequently equilibrated in 1% BSA/PBS for 60 s. Sera were diluted 100-fold in PBS +1% BSA, and binding to the immobilized proteins was assessed for 300 s. The naive prebleed sera response for each group of immunogens was used as a reference.

### Cryo-EM specimen preparation and data collection

S2P spike samples that had not been subjected to low-pH treatment were vitrified at 0.5 mg/ml in PBS. To prepare the low pH-treated specimen, Na-acetate, pH 5.5, was added to the S2P sample that had been stored for 12 days at 4 °C in PBS to a final concentration of 100 mM. After a 10-min incubation at 4 °C, Trizma, pH 8.2, was added to a final concentration of 50 mM. This brought the pH value back to 7.4 as determined by measuring pH after mixing proportional volumes of the same buffers in a separate experiment. The final protein concentration was 0.5 mg/ml. Immediately before vitrification, Quantifoil R 2/2 gold grids were glow-discharged using a PELCO easiGlow device (air pressure: 0.39 mBar, current: 20 mA, duration: 30 s). Cryo-EM specimens were prepared using an FEI Vitrobot Mark IV plunger at 4 °C and 95% humidity; the drop volume was 2.7 μl. Datasets were collected at the National CryoEM Facility (NCEF), National Cancer Institute, using two Thermo Scientific Titan Krios G3 electron microscopes equipped with K3 direct electron detectors and Gatan Quantum GIF energy filters (slit width: 20 eV) (Table S2). For the pH-treated S2P specimen, an additional dataset was collected with the stage tilted to 30° to improve angular coverage; this dataset was merged with the original dataset during computational processing.

### Single-particle analysis of cryo-EM data

Single-particle analysis was performed using the Frederick Research Computing Environment (FRCE) computing cluster (0-day and ∼30-day specimens) and the Biowulf computing cluster (pH-treated specimen). MotionCorr2 was used for patch-based movie frame alignment (38). Contrast transfer function (CTF) parameters were estimated with ctffind4 (39) (FRCE) or GCTF (40) (Biowulf). Particle picking was performed with cryOLO 1.4 (FRCE) or 1.5 (Biowulf) using a general network model (30). The following steps were performed using Relion 3.1 (41) unless otherwise stated. Particles were extracted with 4× binning and subjected to 2D classification. For the ∼30-day dataset, the initial 3D reference was obtained using EMAN 2.1 (42); this 3D reference was used to perform 3D classification for all three datasets. Particles contributing to high-quality 3D classes were re-extracted without binning, followed by an additional round of 3D classification. For the 0-day dataset, this 3D classification produced two distinct conformations, with one (93% of particles) and two (7% of particles) RBDs in the up position; the corresponding subdatasets were then processed separately. Only the one-up conformation was resolved in the remaining two datasets. All the structures were gradually improved with rounds of 3D auto-refinement, CTF refinement, and Bayesian polishing. Local resolution was determined with ResMap 1.1.4 (43). Validation results for the three Cryo-EM reconstructions and a description of the two-up 0-day map are presented in Figs. S5–S8.

RBD occupancy quantification was performed using local 3D classification without particle realignment. A mask encompassing both the up and down conformations of the RBD was first created (Fig. S4*A*). To do this, two copies of the 0-day cryo-EM map were aligned in UCSF Chimera (44) such that RBD A (predominantly up position) of one copy was in register with RBD B (predominantly down position) of the other copy. The densities corresponding to the two RBDs were isolated by segmentation and combined. A soft mask was then created from this composite density. This mask was used to perform local 3D classification into five classes without particle alignment for each RBD of each cryo-EM map. Before evaluating RBD occupancy, equivalent map levels were first determined by aligning the three refined maps (before postprocessing) and visually comparing the density in the stable S2 region. The RBD 3D classes were then examined at these equivalent map levels (0.008 for one-up 0-day map; 0.007 for two-up 0-day map; 0.0068 for the ∼30-day map; 0.0073 for the pH-treated spike map) and categorized as belonging to the up or down conformation or lacking a defined conformation (Fig. S4*B*). The fractions of the up, down, and undefined states were calculated based on the numbers of particles that contributed to each class.

### Atomic model generation

The cryo-EM structure of the S2P spike at pH 5.5 (PDB ID 6xm3) was docked into the one-up 0-day map using UCSF Chimera and refined by alternating rounds of model building in Coot (45) and real-space refinement in Phenix (46). The refined structure was used as a starting model for the ∼30-day and pH-treated S2P spike structures, which were refined in the same way. Structure validation was performed with Molprobity (47). Map-model correlations were evaluated with phenix.mtriage (48). Figures were prepared with UCSF Chimera.

## Data availability

Cryo-EM maps of the freshly purified (0 day), aged (∼30 days), and refolded by low-pH treatment SARS-CoV-2 S2P have been deposited to the EMDB with accession codes EMD-23982, EMD-23983, and EMD-23984, respectively, and fitted coordinates have been deposited to the PDB with accession codes 7MTC, 7MTD, and 7MTE, respectively.

## Supporting information

This article contains supporting information (10, 15, 33, 34, 49, 50, 51).

## Conflict of interest

The authors declare that they have no conflicts of interest with the contents of this article.
