# Supplementary material for: SARS-CoV-2 S2P spike ages through distinct states with altered immunogenicity
Source: J Biol Chem. 2021 Aug 27;297(4):101127. doi: 10.1016/j.jbc.2021.101127 (PMC8393506; doi:10.1016/j.jbc.2021.101127)
Supplement: Supplemental Figures S1–S8 and Tables S1, S2 [file mmc1.pdf]

Table S1: Kinetics of antibody and receptor interaction with spike samples.

Epitope	Antibody	Neutralization (IC50)	Highest concentration S2P (nM)	S2P samples	K_{app} (M)	K_{app} Error	k_{on}(1/Ms)	k_{on} Error	k_{off}(1/s)	k_{off} Error	Ref.
RBD	**Dimeric ACE2-Fc**	5 µg/ml	50	0d	2.57E-10	3.61E-12	2.75E+05	2.01E+03	7.08E-05	8.49E-07	(33, 49)
				8d	<1.0E-12	2.96E-12	4.53E+05	5.34E+03	3.75E-07	1.34E-06	
				8d pH	1.74E-10	3.13E-12	2.15E+05	1.26E+03	3.74E-05	6.36E-07	
				~30d	N/A*						
RBD	**P2B-2F6**	0.05 µg/ml	25	0d	7.05E-11	1.15E-12	9.14E+05	7.07E+03	6.44E-05	9.26E-07	(15)
				8d	6.36E-11	1.53E-12	9.74E+05	1.07E+04	6.20E-05	1.32E-06	
				8d pH	8.87E-11	1.48E-12	6.85E+05	5.24E+03	6.08E-05	9.03E-07	
				~30d	5.26E-10	1.49E-11	2.34E+05	5.29E+03	1.23E-04	2.10E-06	
RBD	**S309**	0.069 µg/ml	50	0d	<1.0E-12	2.66E-12	1.80E+05	8.70E+02	<1.0E-07		(50)
				8d	<1.0E-12	3.08E-12	2.08E+05	1.29E+03	<1.0E-07		
				8d pH	5.27E-11	3.45E-12	1.34E+05	7.14E+02	7.04E-06	4.60E-07	
				~30d	<1.0E-12	1.55E-10	2.16E+04	4.02E+03	<1.0E-07		
RBD	**2-43**	0.003 µg/ml	50	0d	5.70E-10	9.29E-12	2.22E+05	2.58E+03	1.26E-04	1.45E-06	(10)
				8d	9.76E-10	2.66E-11	1.51E+05	3.30E+03	1.47E-04	2.38E-06	
				8d pH	6.22E-10	1.51E-11	2.06E+05	3.58E+03	1.28E-04	2.15E-06	
				~30d	N/A*						
RBD	**B38**	0.177 µg/ml	50	0d	3.62E-10	5.90E-12	2.97E+05	3.18E+03	1.07E-04	1.32E-06	(34)
				8d	4.11E-10	6.08E-12	3.58E+05	4.01E+03	1.47E-04	1.42E-06	
				8d pH	4.15E-10	5.81E-12	2.36E+05	2.08E+03	9.81E-05	1.06E-06	
				~30d	N/A*						
RBD	**CR3022**	NR†	50	0d	<1.0E-12	3.76E-12	1.10E+05	5.95E+02	<1.0E-07		(51)
				8d	4.39E-11	3.33E-12	1.76E+05	1.02E+03	7.74E-06	5.86E-07	
				8d pH	2.04E-10	4.15E-12	9.08E+04	4.79E+02	1.85E-05	3.64E-07	
				~30d	N/A*						
NTD	**2-51**	0.007 µg/ml	25	0d	7.96E-12	1.08E-12	7.00E+05	4.63E+03	5.57E-06	7.58E-07	(10)
				8d	1.73E-11	1.19E-12	6.53E+05	4.43E+03	1.13E-05	7.74E-07	
				8d pH	5.60E-11	1.13E-12	5.51E+05	2.90E+03	3.09E-05	6.02E-07	
				~30d	N/A*						
NTD	**4-18**	0.02 µg/ml	50	0d	<1.0E-12	7.11E-12	1.33E+05	1.54E+03	<1.0E-07		(10)
				8d	3.41E-10	7.60E-12	1.35E+05	1.39E+03	4.58E-05	9.06E-07	
				8d pH	8.26E-11	5.16E-12	1.25E+05	9.76E+02	1.04E-05	6.42E-07	
				~30d	5.01E-09	1.66E-09	7.65E+03	2.50E+03	3.83E-05	2.13E-06	
S2	**s652-112**	NR†	50	0d	1.05E-11	3.15E-12	2.26E+05	1.47E+03	2.37E-06	7.10E-07	(33)
				8d	5.97E-12	2.25E-12	3.30E+05	2.18E+03	1.97E-06	7.42E-07	
				8d pH	5.04E-11	2.36E-12	1.74E+05	7.14E+02	8.78E-06	4.08E-07	
				~30d	N/A*						

† Not Reported: denotes non-neutralizing antibodies or where neutralization IC_{50} has not been reported.
* Not Applicable: denotes kinetics could not be fit with a 1-to-1 model.

Table S2: Cryo-EM data collection, refinement and validation statistics.

	0d<br>EMDB-23982<br>PDB 7MTC	30d<br>EMDB-23983<br>PDB 7MTD	12d refolded<br>EMDB-23984<br>PDB 7MTE
**Data collection and processing**			
Magnification	105,000	105,000	105,000
Voltage (kV)	300	300	300
Electron exposure (e^{-}/Å^2)	40	40	40
Defocus range (μm)	-1.0 to -2.5	-1.0 to -2.5	-1.0 to -2.5
Pixel size (Å)	0.855	0.855	0.873
Symmetry imposed	C1	C1	C1
Initial particle images (no.)	1,937,208	1,117,027	2,314,997
Final particle images (no.)*	403,167	95,641	252,067
Map resolution (Å)	2.6	3.5	3.2
FSC threshold	0.143	0.143	0.143
Map resolution range (Å)	1.9-5.6	2.9-7.2	1.9-6.4
**Refinement**			
Initial model used (PDB code)	6XM3	0d structure	0d structure
Model resolution (Å)	2.7	3.6	3.1
FSC threshold	0.5	0.5	0.5
Map sharpening B factor (Å^2)	-30.0	-70.7	-67.0
Model composition			
Non-hydrogen atoms	21178	18847	20612
Protein residues	2642	2362	2568
Ligands (glycans)	42	42	42
Water	0	0	0
B factors (Å^2)(mean)			
Protein	63.2	94.2	64.7
Ligand	73.9	112.0	78.7
Water	N/A	N/A	N/A
R.m.s. deviations			
Bond lengths (Å)	0.013	0.006	0.004
Bond angles (°)	1.13	0.867	0.804
Validation			
MolProbity score	1.32	1.48	1.30
Clash score	1.53	2.65	2.16
Poor rotamers (%)	0.43	0.34	0.04
Ramachandran plot			
Favored (%)	93.49	93.37	95.51
Allowed (%)	6.51	6.59	4.49
Disallowed (%)	0	0.04	0

* Unfolded molecules were not included in final particle analysis.

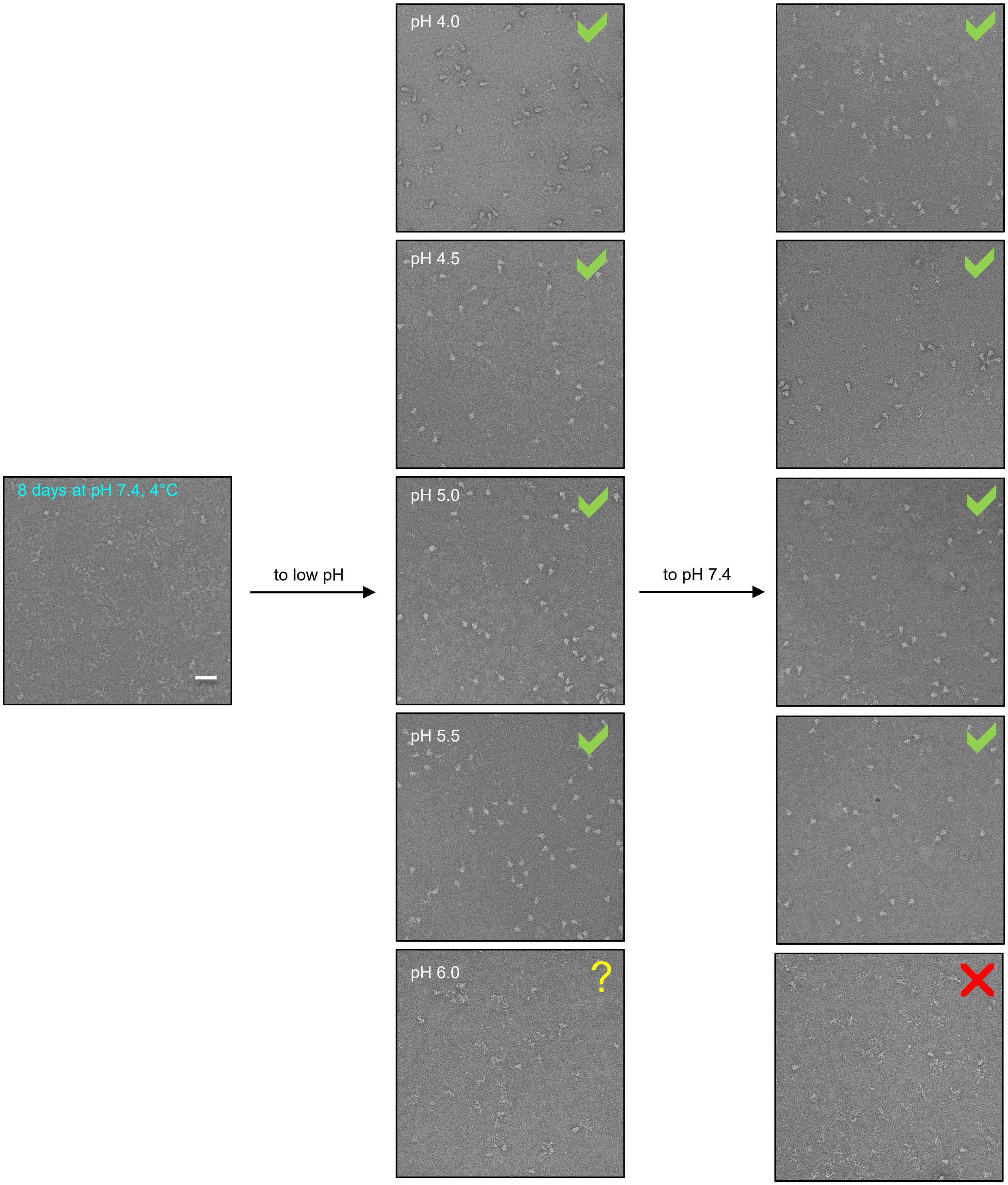

Fig. S1: Refolding of aged spike by low-pH treatment at different pH levels evaluated by NS-EM. S2P spike after 8 days of storage at 4°C, pH 7.4 was transferred to indicated pH for 5 min, returned to pH 7.4 and assessed by negative-stain EM. Reducing pH to 4.0–5.5 fully refolded the S2P spike (indicated by green check marks), and it maintained its folded state at pH 7.4. Reducing the pH to 6.0 partially refolded the S2P spike (yellow question mark), but most trimers were found to be unfolded upon transferring back to pH 7.4 (red “x” symbol). Scale bar: 50 nm.

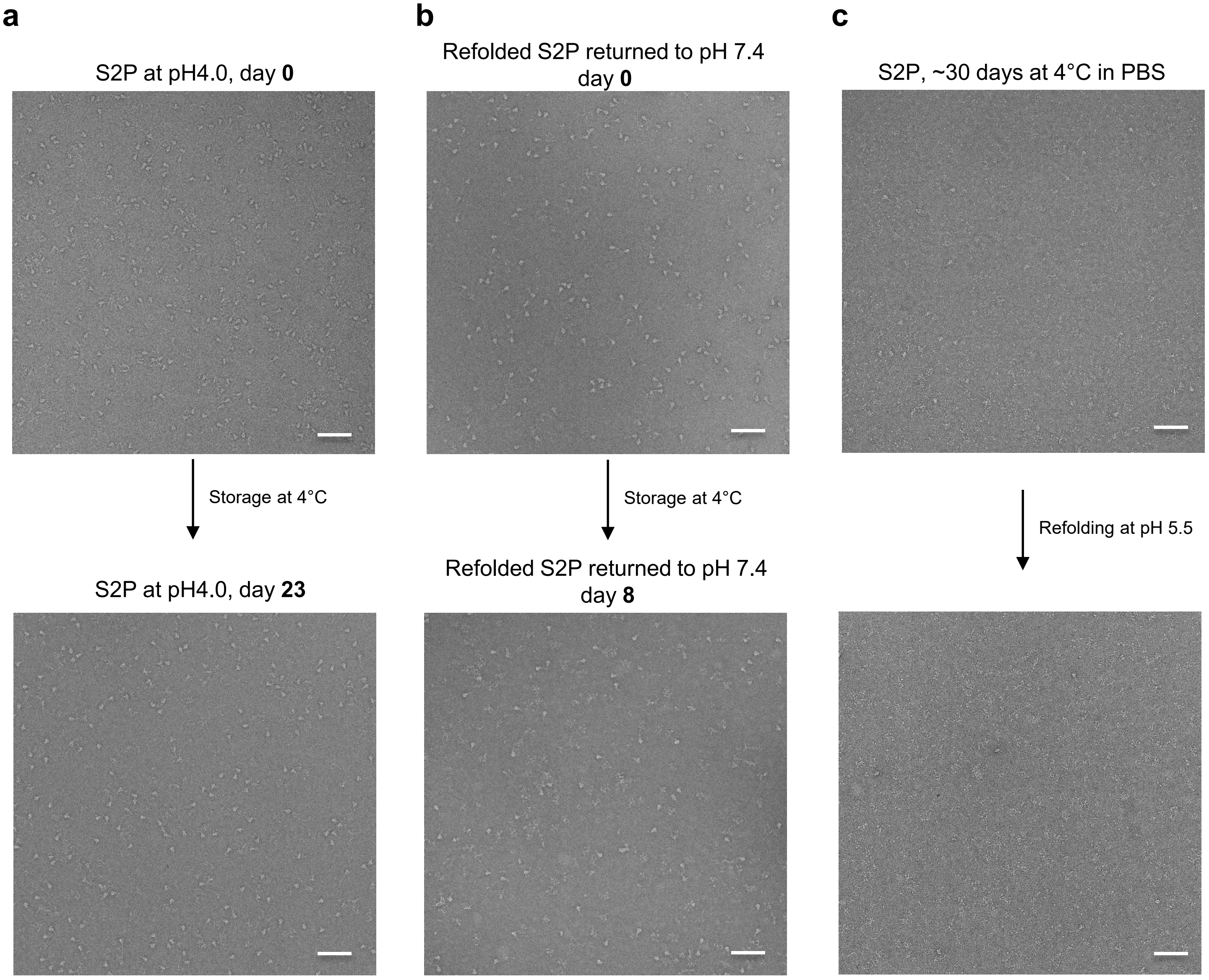

Fig. S2: Storage-related properties of S2P spike revealed by negative-stain electron microscopy (NS-EM).

(a) S2P spike remains stable when stored at low pH. The spike was transferred to 100 mM acetate buffer, pH 4.0, and stored at 4°C. NS-EM was performed at 0 and 23 days of storage. Scale bars: 100 nm. (b) S2P spike refolded by low-pH treatment (pH 5.5) and returned to pH 7.4 gradually unfolds when stored at 4°C. NS-EM was performed at 0 and 8 days of storage at 4°C. Scale bars: 100 nm. (c) Low-pH treatment failed to refold S2P spike after prolonged storage at 4°C. S2P spike that had been stored in PBS at pH 7.4 and 4°C for ~30 days was transferred to pH 5.5. NS-EM revealed no refolding of the spike trimer. Scale bars: 100 nm.

a

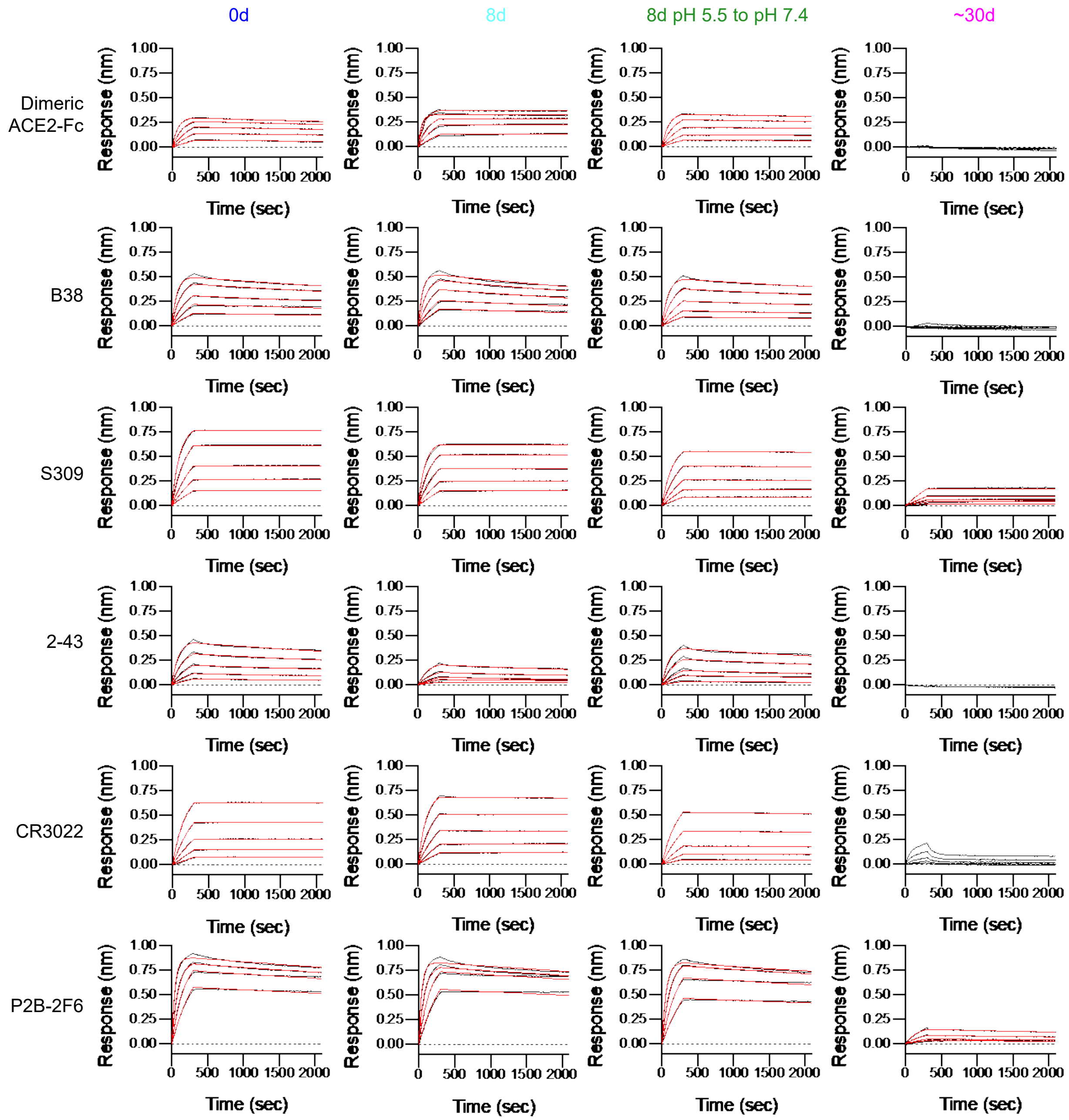

Fig. S3. Biolayer interferometry.
Biolayer interferometry sensorgrams for (a) RBD-tageting antibodies and dimeric ACE2 receptor, (b) NTD-targeting antibodies and (c) S2-targeting antibodies binding 0d, 8d, 8d pH 5.5 to pH 7.4 and ~30d spike samples. Two-fold dilution series of spike samples were assessed, starting at 50nM for dimeric ACE2, B38, S309, 2-43, CR3022, 4-18, S652-112, and 25nM for P2B-2F6, 2-51, 1-69, 5-17. Black lines represent experimental data and red lines show the kinetic fitting to a 1:1 binding interaction model.

(Continued on next page)

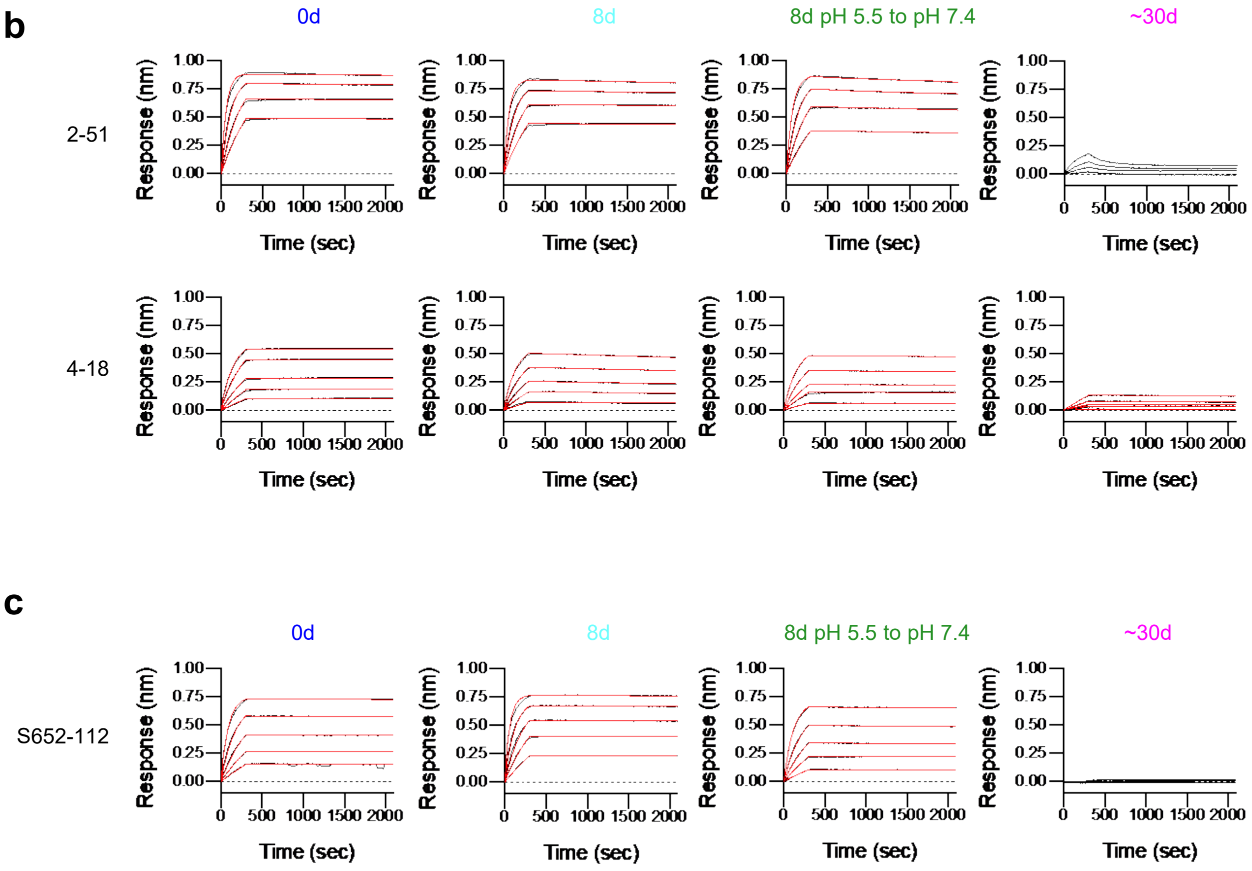

Fig. S3. Biolayer interferometry (cont.)

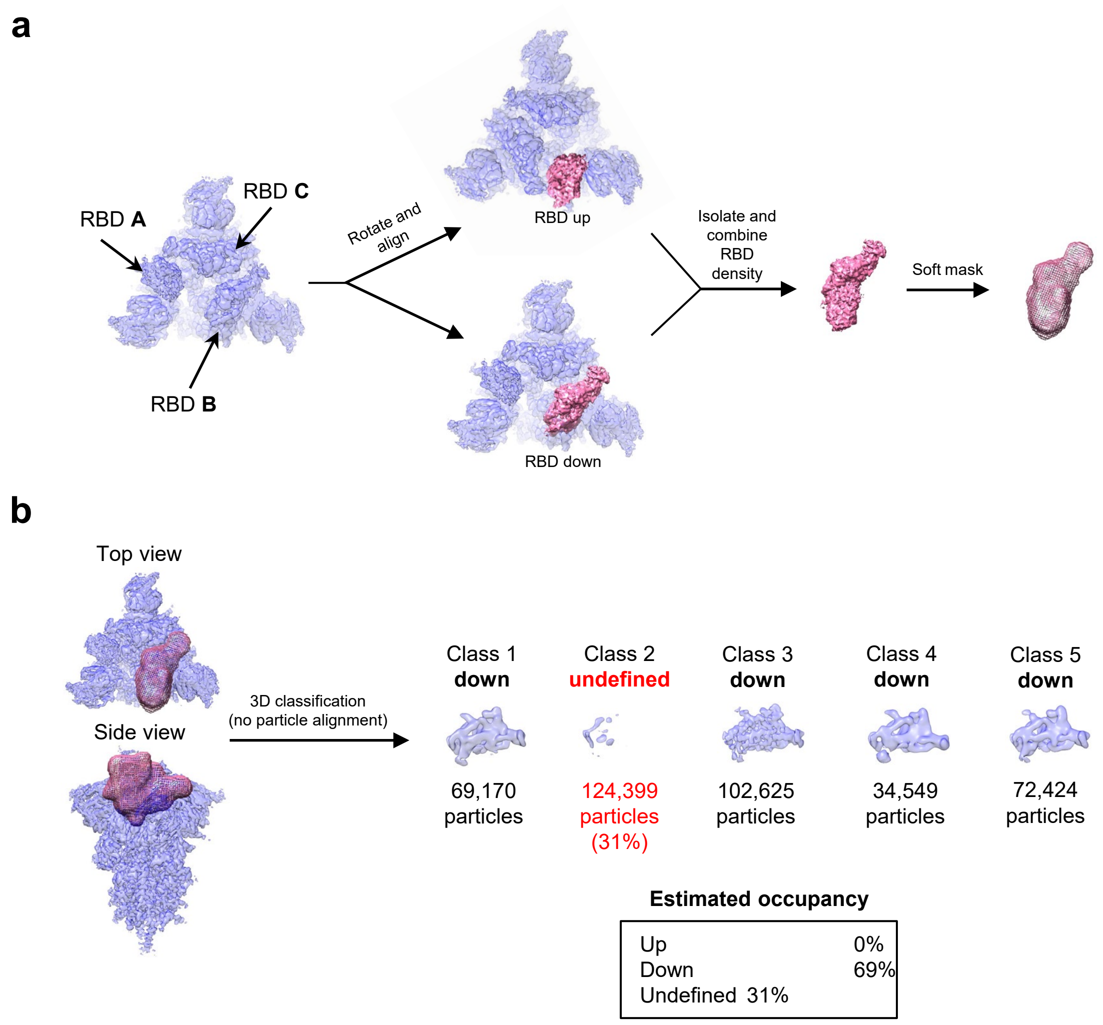

Fig. S4: Quantification of RBD occupancy in the cryo-EM maps.
(a) Approach to creating masks for local 3D classification. The RBDs in the up and down position are superposed by aligning two copies of the map, and the corresponding densities are extracted and combined. A soft composite mask is then generated. (b) Example of RBD occupancy quantification (freshly purified protein, RBD B) using the mask created in (a).

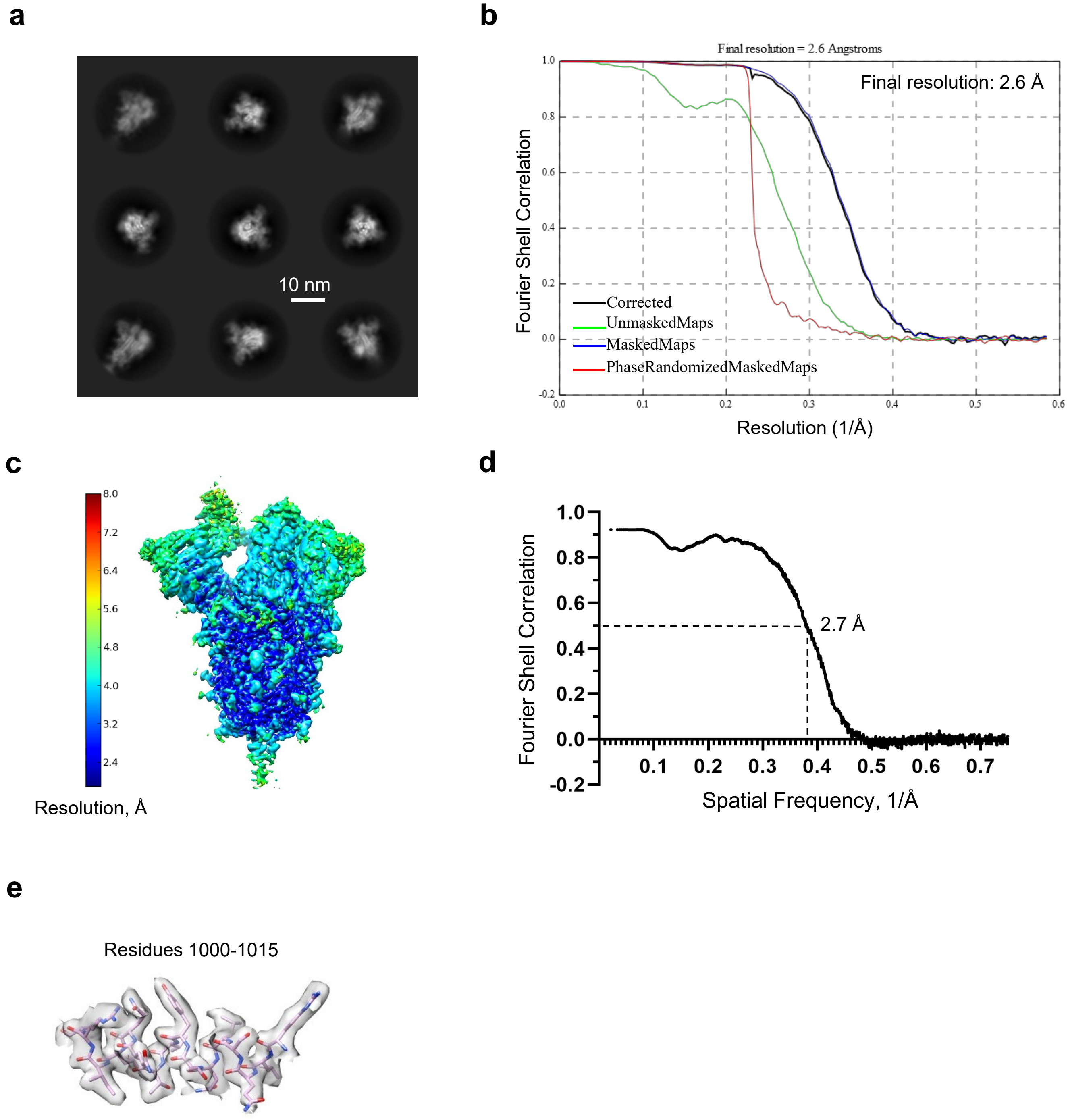

Fig. S5: Validation of the cryoEM map and atomic model of the freshly purified (0d) S2P spike with one RBD in the up position, as generated from 403,167 (93%) particles.

(a) Representative high-resolution 2D class averages. (b) Gold-standard resolution data generated by Relion. At the 0.143 threshold, the resolution is 2.6 Å. (c) Results of local resolution analysis using ResMap. The map is colored according to local resolution. (d) Fourier shell correlation curve between the map and the atomic model. (e) Example of cryo-EM density.

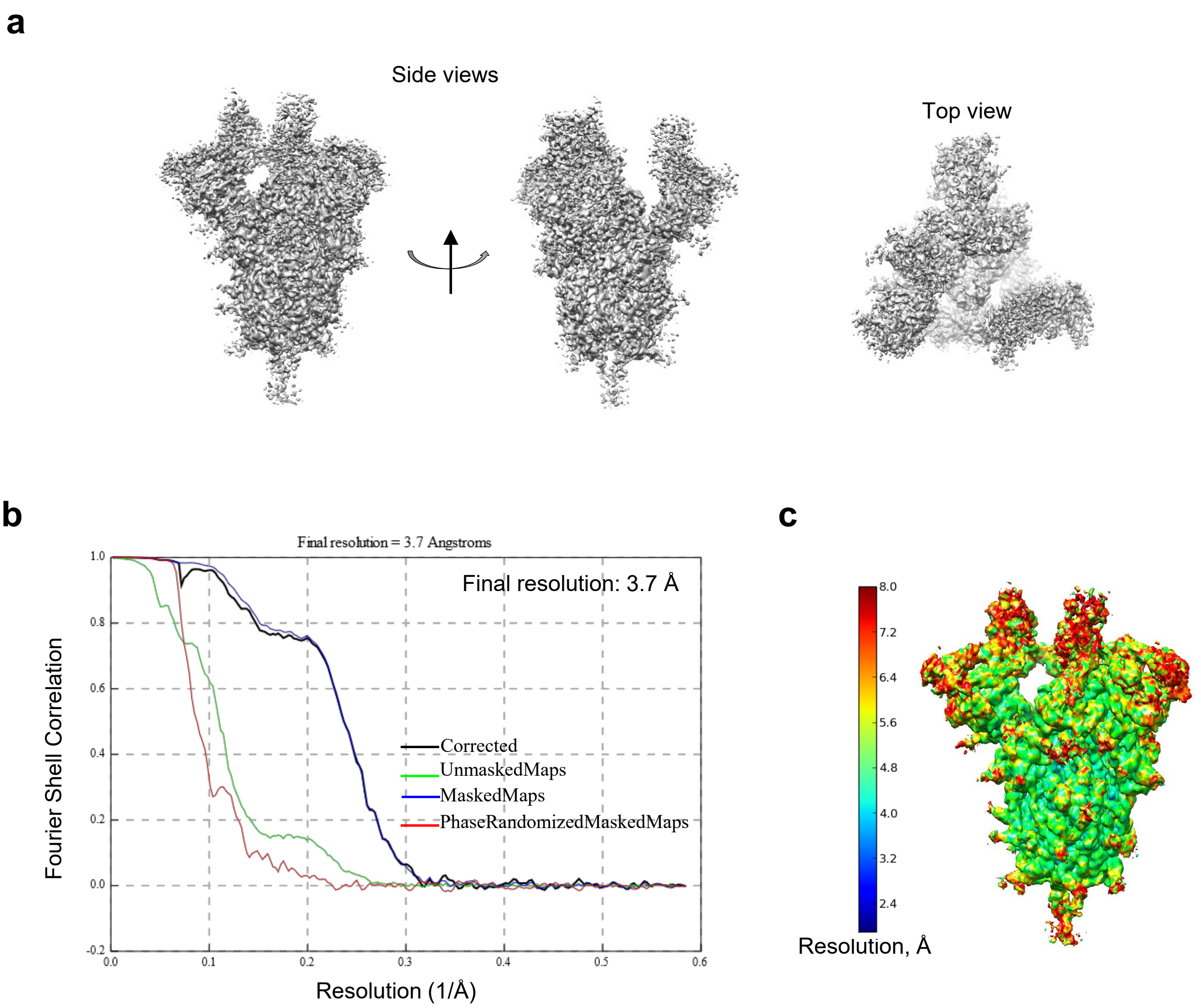

Fig. S6: CryoEM structure of the freshly purified (0d) S2P spike with two RBDs in the up position, generated from 28,379 (~7%) of particles.

(a) Representative views of the map. (b) Gold-standard resolution data generated by Relion. At the 0.143 threshold, the resolution is 3.7 Å. (c) Results of local resolution analysis using ResMap. The map is colored according to local resolution.

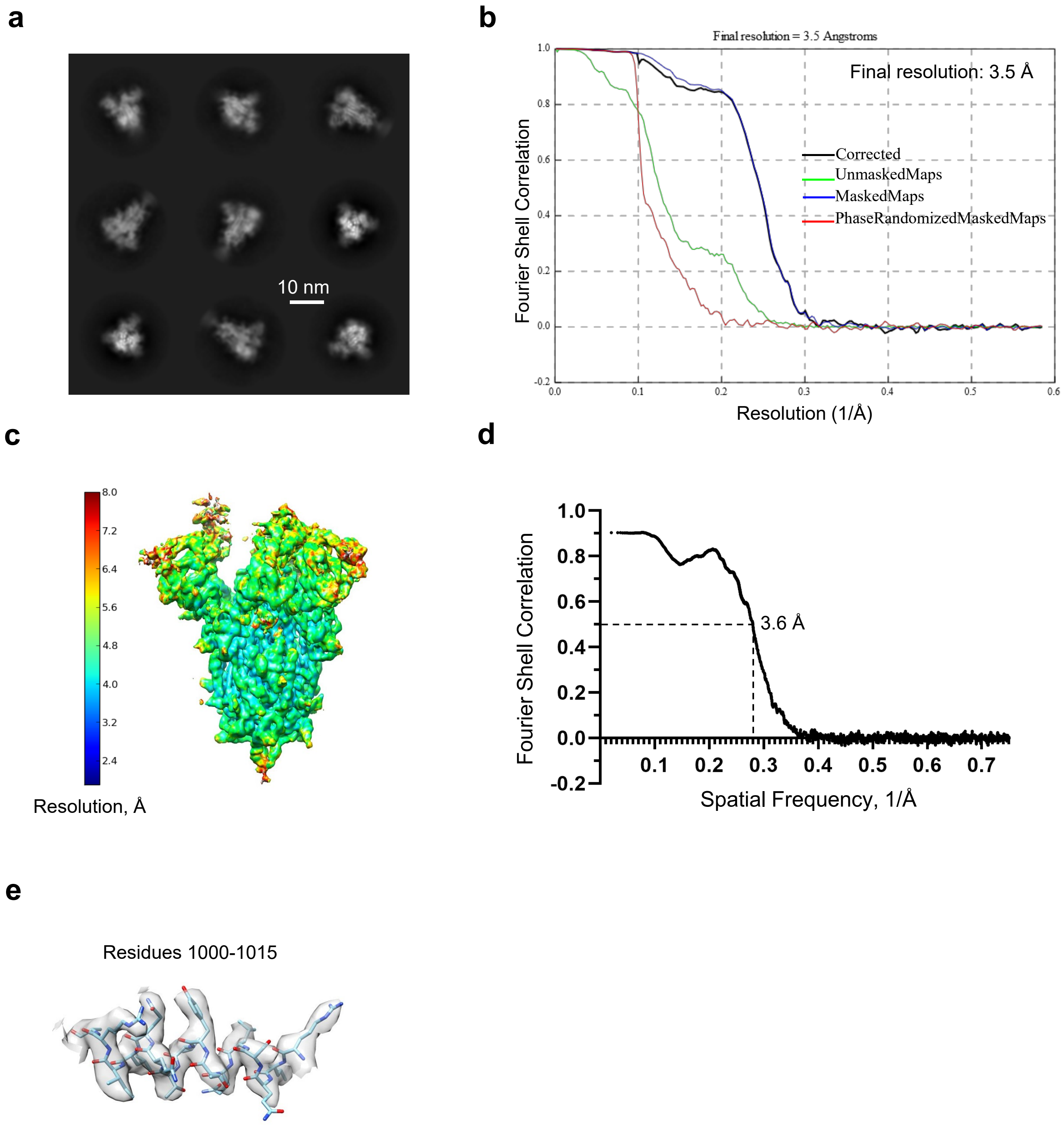

Fig. S7: Validation of the cryoEM map and atomic model of the "aged" (~30d) S2P spike.
(a) Representative high-resolution 2D class averages. (b) Gold-standard resolution data generated by Relion. At the 0.143 threshold, the resolution is 3.5 Å. (c) Results of local resolution analysis using ResMap. The map is colored according to local resolution. (d) Fourier shell correlation curve between the map and the atomic model. (e) Example of cryo-EM density.

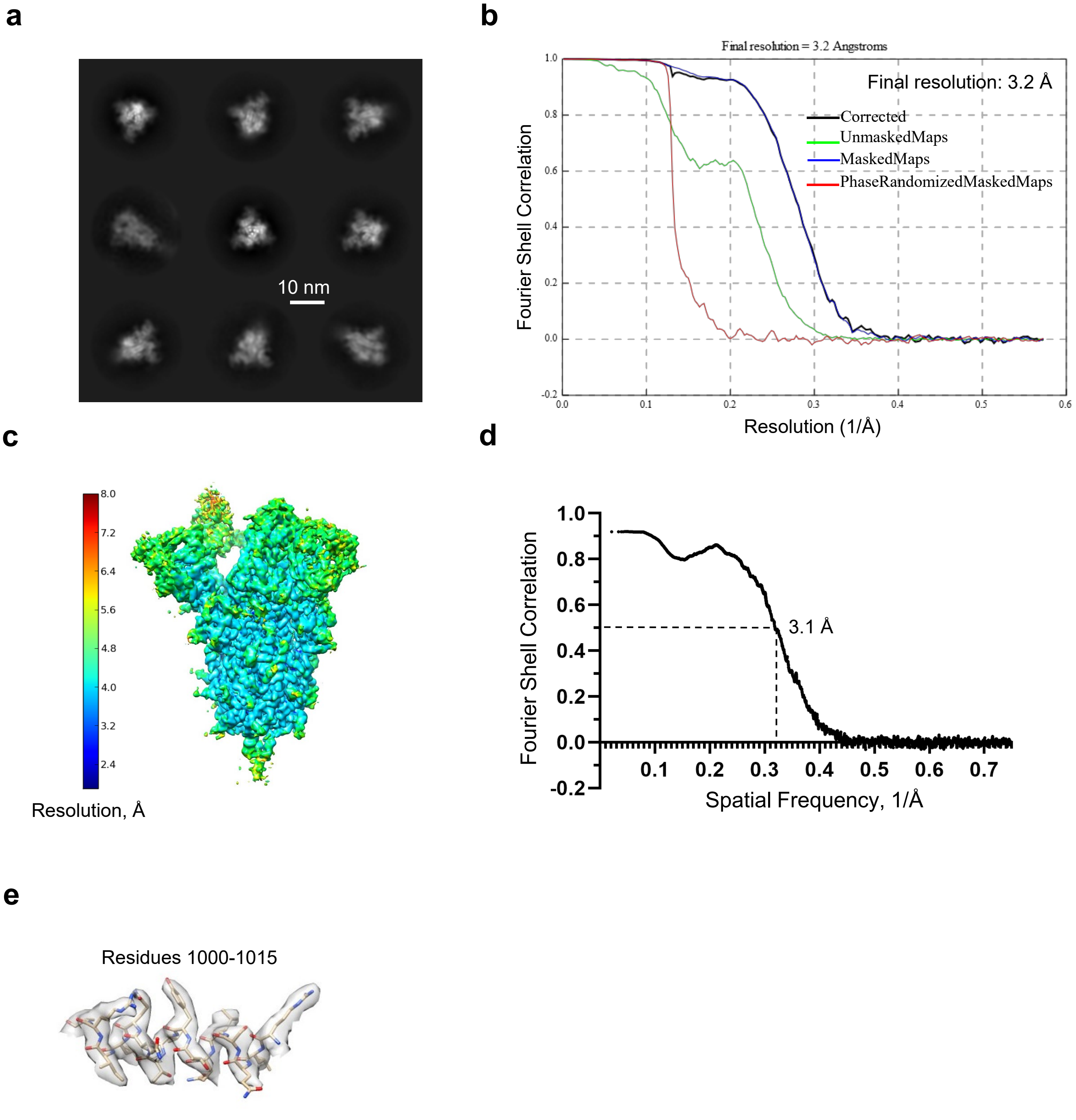

Fig. S8: Validation of the cryoEM map and atomic model of the low pH-treated S2P spike.
(a) Representative high-resolution 2D class averages. (b) Gold-standard resolution data generated by Relion. At the 0.143 threshold, the resolution is 3.2 Å. (c) Results of local resolution analysis using ResMap. The map is colored according to local resolution. (d) Fourier shell correlation curve between the map and the atomic model. (e) Example of cryo-EM density.
